# Dual NDP52 Function in Persistent CSFV Infection

**DOI:** 10.3389/fmicb.2019.02962

**Published:** 2020-01-08

**Authors:** Shuangqi Fan, Keke Wu, Chaowei Luo, Xin Li, Mengpo Zhao, Dan Song, Shengming Ma, Erpeng Zhu, Yuming Chen, Hongxing Ding, Lin Yi, Jun Li, Mingqiu Zhao, Jinding Chen

**Affiliations:** College of Veterinary Medicine, South China Agricultural University, Guangzhou, China

**Keywords:** classical swine fever virus, NDP52, ubiquitination, CD63, NF-κB

## Abstract

Viruses have evolved many mechanisms to escape host antiviral responses. Previously, we found that classical swine fever virus (CSFV) infection induces autophagy using the autophagosome as a self-replication site, thereby evading the host immune response and promoting long-term infection. However, the underlying mechanisms used by CSFV to enter autophagosomes and the mechanism by which autophagy promotes viral replication remain unclear. We found that CSFV infection inhibited autophagy receptor nuclear dot protein 52 kDa (NDP52) expression, ubiquitination, and SUMO2-4 modification. Further analyses revealed that CSFV mediated ubiquitination and SUMOylation of NDP52 via Pten-induced kinase 1 (PINK1)-Parkin. Moreover, NDP52 inhibition also inhibited CSFV replication and the induction of mitophagy marker proteins expression. Inhibition of NDP52 reduced CD63 expression and binding to CSFV E2 protein, which has an essential role in persistent CSFV infection. As NDP52 has a close relationship with the NF-κB innate immunity pathway and plays an important role in the antiviral response, we investigated whether NDP52 inhibited CSFV replication through the release of immune factors and antivirus signals. Our results showed that inhibiting NDP52 boosted interferon and TNF release and promoted NF-κB pathway activation. In summary, we found that NDP52 inhibition not only reduces CSFV binding and entry into autophagic vesicles, but also inhibits CSFV replication by active NF-κB antiviral immune pathways. Our data reveal a novel mechanism by which NDP52, an autophagy receptor, mediates CSFV infection, and provide new avenues for the development of antiviral strategies.

## Introduction

Swine fever, caused by classical swine fever virus (CSFV) infection, is characterized by acute fever and death, and is classified as a Class A infectious disease by the World Organization for Animal Health (OIE) (Paton and Greiser-Wilke, 2003). CSFV belongs to the genus *Pestivirus* within the family Flaviviridae. The virus has a small, enveloped, single-stranded, positive-sense 12.3 kb RNA genome with a long, open reading frame that encodes a 3898 amino acid polypeptide (Becher et al., 2003). Co-and post-translational processing of the polypeptide by cellular and viral proteases yields 12 cleavage products, including four structural proteins (C, Erns, E1, and E2) and eight non-structural proteins (N^pro^, p7, NS2, NS3, NS4A, NS4B, NS5A, and NS5B) (Heinz-Jurgen et al., 1991). CSFV can infect several cells types, including immune cells, leading to cellular immunosuppression (Fan et al., 2018). However, CSFV infection does not cause typical pathological changes, and the underlying infection mechanisms remain unclear (Bensaude, 2004; Johns et al., 2009).

Macroautophagy, hereafter referred to as autophagy, is an internal balancing mechanism for maintaining homeostasis in eukaryotic cells. After receiving an autophagy induction signal, such as pathogen infection (Deretic et al., 2013), starvation (Tattoli et al., 2012), growth factor withdrawal (Lum et al., 2005), endoplasmic reticulum (ER) stress (Ciechomska et al., 2013), or oxidative stress (Scherz-Shouval et al., 2007), the cell forms a small liposome-like membrane structure in the cytosol, which expands to form a bowl-like structure consisting of two layers of lipid bilayers that can be observed under electron microscopy. The bowl structure is called a phagophore. Many components in the cytoplasm, including protein aggregates, damaged organelles, and foreign invading pathogens, are wrapped in vesicles and then closed into a closed spherical autophagosome (Høyer-Hansen and Jäättelä, 2008). Microtubule-associated protein 1 light chain 3 (MAP1LC3 or simply LC3), consisting of the interconvertible forms LC3-I and LC3-II, is involved in the formation of autophagosome membranes. Early pro-LC3 cleavage by ATG4 exposes the C-terminal glycine to form the cytosolic soluble form LC3-I, which is modified by ubiquitination and coupled with the substrate PE on the surface of the autophagosome membrane under the action of the E1-like enzyme ATG7, the E2-like enzyme ATG3, and the E3-like enzyme ATG5-ATG12-ATG16L complex to form the membrane-bound form LC3-II. After autophagosome formation, this fuses with lysosomes to form autolysosomes. Under the action of various hydrolases, the substrate in the autophagosomes is degraded (Bizargity and Schröppel, 2014). Autophagy is a way for cells respond to unfavorable environmental factors. Many RNA viruses, such as enteroviruses, hepatitis C virus (HCV), and CSFV, circumvent and utilize host autophagic machinery to promote viral propagation (Pei et al., 2013; Mohamud and Luo, 2018; Wang and Ou, 2018). During CSFV infection, the viral proteins NS5A and E2 colocalize with the autophagy marker CD63 on autophagosome-like vesicle membranes. Moreover, CSFV infection can use mitophagy to inhibit cell apoptosis to create a persistent environment for viral infection (Pei et al., 2016; Gou et al., 2017). However, the mechanisms underlying CSFV-autophagosome entry are unclear.

Autophagy was initially considered to be non-selective, but recent studies have found that autophagy can also be selective. The most important feature of the selective autophagy pathway is the involvement of autophagy receptors that recognize and transport autophagic substrates, thereby regulating autophagy substrate degradation under very precise dynamic control (Lazarou et al., 2015). These autophagy receptors contain a conserved LC3-interacting region (LIR) domain, which binds to Atg8 molecules on autophagosomes and mediate autophagy degradation (Zaffagnini and Martens, 2016). Ubiquitin acts as a signaling molecule, inducing polyubiquitination of autophagy substrates (Kocaturk and Gozuacik, 2018). Autophagy receptor proteins recognize and bind autophagic substrates in a UBA domain-dependent or -independent manner. The LIR is anchored to the autophagosome membrane, followed by autophagosome fusion, lysosome fusion, and substrate degradation in lysosomes (cargo recognition and trafficking in selective autophagy) (Shaid et al., 2012; Nakamura and Yoshimori, 2017). In addition to the autophagy receptor functions, the protein itself is also ubiquitinated. Hou et al. found that the ubiquitin ligase HACE1 with tumor suppressor activity binds to the OPTN protein and catalyzes the polyubiquitination of OPTN. OPTN is modified by HACE1 with a K48-linked polyubiquitin chain, followed by autophagy–lysosomal pathway degradation. HACE1 mediates the modification of the 193th lysine of OPTN by a ubiquitin chain, which specifically interacts with the UBA domain of p62 to activate autophagy. Clearly, ubiquitinated OPTN interacts with p62 to increase the efficiency of autophagy receptor-mediated transport of substrates to autophagic vacuoles, significantly increasing autophagic flux and thereby more effectively recruiting and transporting autophagy substrates (Liu et al., 2014).

It is well known that NF-κB plays a key role in regulating the immune response to infection. It participates in the inflammatory response and immune response and regulates apoptosis and the stress response (Liu et al., 2017). There are three main signal transduction pathways that activate NF-κB: the classical pathway, bypass pathway, and atypical pathway (Oeckinghaus and Ghosh, 2009). Several proteins encoded by NF-κB target genes are involved in the activation of immune and inflammatory responses. NF-κB activation during viral infection is interpreted as a host protective response to viral pathogens (Schmitz et al., 2014). Type I interferons (IFNs) also play an important role in the immune response to viruses. The production of type I IFN in immune cells is mediated by pattern recognition receptors in the host cell (Ivashkiv and Donlin, 2013). There are four main ways to induce the production of type I IFN: (1) DNA virus activates the second messenger cGAMP (cyclic GMP-AMP) induction pathway; (2) RNA virus activates the RIG-I-like receptor (RLR) induction pathway; (3) the Toll-like receptors TLR3 and TLR4 activate the adaptor protein TRIF induction pathway; and (4) TLR7/TLR8 and TLR9 activate the transcription factor IRF7 induction pathway (Majzoub et al., 2019). Interestingly, CSFV replication in cells suppresses type I IFN-inducible antiviral activity and apoptosis by interfering with IFN production, resulting in the persistent survival of CSFV in host cells *in vitro*.

Nuclear dot protein 52 kDa (NDP52), also called CALCOCO2, has been well studied in xenophagy and primary adaptation to Parkin-mediated mitophagy (Sharma et al., 2018; Ravenhill et al., 2019). Pten-induced kinase 1 (PINK1) can promote NDP52 recruitment and ubiquitination. Further, the xenophagy kinase TBK1 forms a complex with NDP52, thus promoting xenophagy (Fu et al., 2018). In *Salmonella typhimurium* infection, NDP52 promotes pathogen-containing autophagosome maturation and independently regulates targeting of bacteria to mature autophagosomes (Verlhac et al., 2015a). Further studies have found that Rab35 GTPase and myosin VI play important roles in NDP52-microorganism binding and autophagosome maturation (Minowa-Nozawa et al., 2017). During viral infection, NDP52 not only interacts with viral proteins, but also activates RIG-I and NF-κB signaling pathways to exert antiviral effects (Jin et al., 2017). Many studies have shown that NDP52 has a negative regulatory effect on the NF-κB pathway. Moreover, in CVB virus infection, CALCOCO2, but not SQSTM1, suppresses antiviral type I IFN signaling by promoting autophagy-mediated degradation of the mitochondrial antiviral signaling (MAVS) protein (Mohamud et al., 2018). Therefore, we sought to explore the regulatory effect of NDP52 on NF-κB and IFN in swine fever virus infection and hypothesized that it plays an important role in CSFV infection.

Here, we found that CSFV infection activates the PINK1-Parkin pathway, resulting in NDP52 SUMOylation, which inhibits NDP52 and permits CSFV replication. Further, NDP52 colocalizes with viral protein E2, thereby inhibiting CD63 expression, promoting CSFV binding by NDP52 and increasing cytokine release and NF-κB signaling activation.

## Materials and Methods

### Cells, Viruses, and Virus Titration Assays

The swine kidney cell line PK-15 (ATCC, CCL-33) was grown in Dulbecco’s modified Eagle’s medium (DMEM) supplemented with 10% fetal bovine serum (FBS) at 37°C in a 5% CO_2_ incubator. The CSFV strain (Shimen) used in the study was propagated in PK-15 cells. Viral titers in CSFV-infected cell culture media were determined as previously described (Hongchao et al., 2017). Briefly, cells cultivated in 96-well plates were inoculated with 10-fold serial dilutions of virus and incubated at 37°C for 3 days. Cells were fixed with 80% acetone at –20°C for 30 min, and viruses were detected by immunofluorescence assay using mouse anti-CSFV E2 antibody and FITC-conjugated goat anti-mouse secondary antibody. Viral titers are expressed as 50% tissue culture infective doses (TCID50)/0.1 ml.

### Reagents and Antibodies

The chemical reagents MG-132 (M8699) and BAY (11-7082, S2913) were purchased from Sigma-Aldrich and Selleck. The following primary antibodies were used in this study: rabbit polyclonal anti-PARK2 (Abnova, PAB0714), rabbit polyclonal anti-LC3B (Cell Signaling, 2775), rabbit polyclonal anti-MFN2 (Santa Cruz, sc-50331), goat polyclonal anti-VDAC1 (Santa Cruz, sc-32063), goat polyclonal anti-TOM20 (Santa Cruz, sc-11021), mouse monoclonal anti-Beclin-1 (Cell Signaling, 2A4), rabbit polyclonal anti-CD63 (Beyotime, AF1471), mouse monoclonal anti-Ub(A-5) (Santa Cruz, sc-166553), mouse monoclonal anti-CSFV E2 (JBT, 9011), mouse monoclonal anti-IkBa (Cell Signaling, 112B2), mouse monoclonal anti-kB-Ras2 (Santa Cruz, sc-374311), rabbit monoclonal anti-P65 (Santa Cruz, sc-AF1870), mouse monoclonal anti-GAPDH (Beyotime, AG019), mouse monoclonal anti-tubulin (Beyotime, AT819), normal rabbit IgG (Beyotime, A7016), and normal goat IgG (Beyotime, A7007). The polyclonal anti-CSFV Npro was kindly provided by Dr. Xinglong Yu (Veterinary Department, Hunan Agricultural University, China). The secondary antibodies used for immunofluorescence were Alexa Fluor 350 goat anti-mouse IgG (Beyotime, A0412), Alexa Fluor 488 goat anti-mouse IgG (Beyotime, A0428), and Alexa Fluor 647 goat anti-rabbit IgG (Beyotime, A0468). The secondary antibodies used for immunoblotting analysis were HRP-conjugated goat anti-mouse IgG (Bioworld Technology, BS12478), HRP-conjugated goat anti-rabbit IgG (Bioworld Technology, BS13278), and HRP-conjugated rabbit anti-goat IgG (Bioworld Technology, BS30503).

### Plasmids and RNA Interference

The EGFP-LC3 plasmid was prepared in our laboratory. Plasmid pAT016 (p-mito-mRFP-EGFP) was a kind gift from Dr. Andreas Till (University of California, United States). Parkin-targeting shRNAs and scrambled shRNA were obtained from Cyagen. Small interfering RNAs (siRNAs) for NDP52 were synthesized by Sangon Biotech. The shRNA and siRNA sequences are listed in Table 1. PK-15 cells grown to 60% confluence in six-well cell culture plates were transfected with siRNA and shRNA using Lipofectamine 3000 reagent (Thermo Fisher, L3000015). Targeted protein knockdown was evaluated by western blotting.

**TABLE 1:** shRNA and siRNA sequences of targeted genes.

**Gene**	**Sequence (5′∼3′)**
shParkin	GCATCACCTGTACGGACATTCTCTTGAAATGTCCGTACAG GTGATGC
scrambled	GCGCGCTTTGTAGGATTCGTTCAAGAGACGAATCCTACAAA GCGCGC
siNDP52	Sense: GCAGGAAGUCCAGUUCAAATT
	Antisense: UUUGAACUGGACUUCCUGCTT
siNC	Sense: UUCUCCGAACGUGUCACGUTT
	Antisense: ACGUGACACGUUCGGAGAATT

### Virus Infection

Twenty-four hours after siNC or siRNA transfection, cells were infected with CSFV at a multiplicity of infection (MOI) of 0.1. Two hours later, the viral inoculum was removed and the infected cells were washed twice with phosphate-buffered saline (PBS) (pH 7.4). DMEM containing 2% FBS was then added to each culture. At various time points post-infection, cell-free culture supernatants and cell lysates were harvested and stored at −80°C until use.

### Immunoprecipitation

For immunoprecipitation analysis, PK-15cells were infected with CSFV at an MOI of 0.1 for 24 h. SUMOylated NDP52 and ubiquitinated Parkin or NDP52 from whole cell lysates (WCL) were incubated on ice with IP lysis buffer (Beyotime, P0013) containing 1 mM PMSF (Beyotime, ST506) for 10 min. The precipitates were removed by centrifugation at 14,000 × *g* for 10 min at 4°C. The supernatant was immunoprecipitated with the appropriate antibodies (anti-Parkin or anti-NDP52) and protein A + G Sepharose (7sea biotech, P001-2). The immunoprecipitated proteins were then analyzed by western blotting with SUMO or ubiquitin antibodies.

### Quantitative Real-Time RT-PCR (qPCR)

For targeted gene expression analysis, total RNA was prepared using a total RNA Kit I (Omega, R6834-01). Complementary DNA (cDNA) was synthesized using PrimeScript RT Master Mix (Takara, RR036A). Real-time qPCR was performed using SYBR Premix Ex Taq II (Takara, RR820A) using an iQ5 iCycler detection system (Bio-Rad, United States). Relative mRNA expression was assessed using the 2^–ΔΔ*Ct*^ method and normalized to the housekeeping gene GAPDH. The primers used are described in Table 2. For virus copy detection, viral RNA was extracted using a MiniBEST Viral RNA/DNA Extraction Kit Ver.5.0 (Takara, 9766) and reverse-transcribed using PrimeScript RT Master Mix (Perfect Real Time; Takara, RR036A). The resulting cDNA was then amplified using SYBR Premix Ex Taq (Tli RNaseH Plus; Takara, RR420B) and an iQ5 iCycler detection system (Bio-Rad, United States). Primer sequences targeting the CSFV NS5B gene were: CSFV1: CCTGAGGACCAAACACATGTTG; CSFV2: TGGTGGAAGTTGGTTGTGTCTG. Viral copy number was calculated using a standard curve from a recombinant plasmid containing the CSFV NS5B gene.

**TABLE 2:** Primers used in this study.

**Gene**	**Sequence (5′∼3′)**	**GenBank**
NDP52	F: CGGAATTCCATGAGGGGCGGGCCCCG R: GGGGTACCTCACAGGTCCTTCAGATCCTT	XM003131552.4
IFNA	F: CTCAGCCAGGACAGAAGCA R: TCACAGCCCAGAGAGCAGA	NM_214393.1
IFNB1	F: TCGCTCTCCTGATGTGTTTCTC R: AAATTGCTGCTCCTTTGTTGGT	NM_001003923.1
TNF	F: TGGCCCAAGGACTCAGATCAT R: TCGGCTTTGACATTGGCTACA	EU682384
GAPDH	F: TGGAGTCCACTGGTGTCTTCAC R: TTCACGCCCATCACAAACA	NM_001206359.1

### Western Blot Analysis

After treatment, cells were washed with cold PBS and incubated on ice with RIPA lysis buffer (Beyotime, P0013B) supplemented with 1 mM PMSF (Beyotime, ST506) for 10 min. Cell lysates were centrifuged at 14,500 × *g* for 20 min at 4°C. Protein concentration was determined using a BCA protein assay kit (Beyotime, P0012). Samples with equal protein amounts were diluted in 5× SDS-PAGE loading buffer and boiled for 5 min. Proteins (20 mg) were separated by SDS-PAGE and transferred onto polyvinylidene fluoride membranes (Beyotime, FFP30). After blocking with PBS containing 2% non-fat milk powder and 0.05% Tween 20 (Sigma-Aldrich, P2287) for 2 h at 25°C, the membrane was incubated with primary antibodies overnight at 4°C. Then, membranes were incubated with corresponding HRP-conjugated secondary antibodies at 37°C for 2 h at appropriate dilutions. The protein bands were visualized using an ECL Plus kit (Beyotime, P0018). Blots were imaged with a CanoScan LiDE 100 scanner (Canon, Japan) and quantified with Image Pro Plus 6.0 software.

### Confocal Microscopy

Cells were grown in 35 mm glass-bottom petri dishes (NEST, GBD-35-20). The indicated interfering RNA was transfected at various time points. Cells were washed with PBS, fixed with 4% paraformaldehyde for 30 min, and washed with 0.2% Triton X-100 (Sigma-Aldrich, T8787) in PBS for 10 min. The cells were blocked in PBS containing 5% bovine serum albumin (BSA; Beyotime, ST023) for 30 min. Next, the cells were stained with primary antibodies and appropriate secondary antibodies for 1 h at 37°C. Wherever indicated, nuclei were stained with DAPI (Beyotime). Fluorescence was visualized with a TCS SP2 confocal fluorescence microscope (Leica).

### Cell Viability Assay

Cell viability was detected by the CCK8 assay according to the manufacturer’s instructions (Dojindo, CK04). In brief, PK-15 cells were cultivated in 96-well plates at a density of 1 × 10^4^ cells per well and cultured for 24 h at 37°C. The cells were transfected with siNDP52, siNC, shParkin or non-targeting shRNA using Lipofectamine 3000 reagent. After 48 h, the medium was replaced with 100 μl of fresh medium containing 10 μl of CCK8. The cells were further cultured for 1 h at 37°C, and the optical density was measured at 570 nm using a model 680 microplate reader (Bio-Rad).

### Statistical Analysis

Statistical analysis was performed with unpaired Student’s *t*-tests, as implemented in GraphPad Prism 5 software (mean ± SD; *n* = 3; ^∗^*P* < 0.05; ^∗∗^*P* < 0.01; ^∗∗∗^*P* < 0.001; ^#^*P* > 0.05).

## Results

### CSFV Infection Inhibits NDP52 (CALCOCO2) Ubiquitination and SUMOylation

**FIGURE 1:**
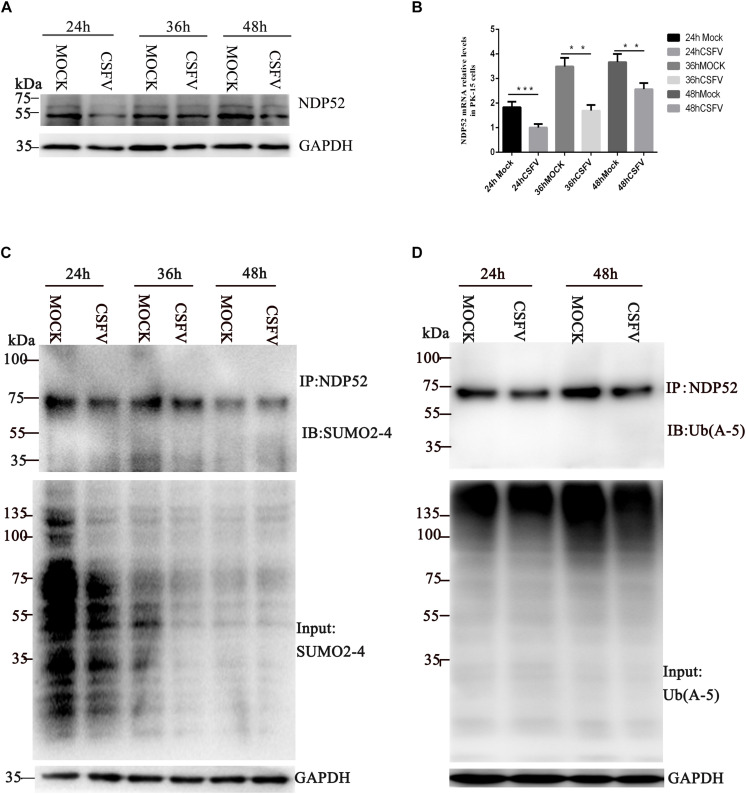
Classical swine fever virus (CSFV) inhibits NDP52 (CALCOCO2) ubiquitination and SUMOylation. **(A)** PK-15 cells were infected with CSFV (MOI = 0.1) or un-infected (MOCK) at the indicated time points. CALCOCO2, N^*pro*^, and GAPDH levels were analyzed by Western blot. **(B)** After CSFV infection, CALCOCO2 mRNA was detected by qRT-PCR. The data represent the mean ± SD of three independent experiments. Data were tested by one-way ANOVA with Fisher’s least significant difference (LSD) *post hoc* correction. ^∗^*P* < 0.05; ^∗∗^*P* < 0.01; ^∗∗∗^*P* < 0.001; #*P* > 0.05. **(C)** PK-15 cells were infected with CSFV. NDP52 SUMOylation was analyzed by immunoblot using anti-SUMO2-4 antibody. SUMO2-4 expression in CSFV-infected cells was used as the input control. **(D)** Ubiquitinated NDP52 was immunoblotted with anti-Ub(A-5) antibody. Ub(A-5) expression in CSFV-infected cells was used as the input control. GAPDH was used as the internal loading control.

To explore whether CSFV infection affects NDP52, we first evaluated changes in NDP52 mRNA and protein expression after CSFV infection. PK-15 cells were tested at several time points post-CSFV infection. NDP52 mRNA and protein expression was decreased by CSFV infection at 24, 36, and 48 h post-infection (hpi) (Figures 1A,B). These results suggest that CSFV infection has a negative effect on the expression of NDP52. Several reports indicate that autophagy receptors undergo self-modification (Liu et al., 2014). Therefore, we examined NDP52 ubiquitination and SUMOylation after CSFV infection. CSFV-infected cell lysate was immunoprecipitated with an NDP52-specific antibody and immunoblotted with SUMO2-4 or Ub(A-5) antibody. These results showed that NDP52 ubiquitination and SUMOylation were decreased in CSFV-infected cells compared to that in un-infected PK-15 cells (Figures 1C,D). The above results indicate that CSFV infection not only inhibits the expression of NDP52, but also mediates the protein modification of NDP52.

### CSFV Infection Promotes Ubiquitination of Parkin and Mediates NDP52 Modification via PINK1-Parkin

**FIGURE 2:**
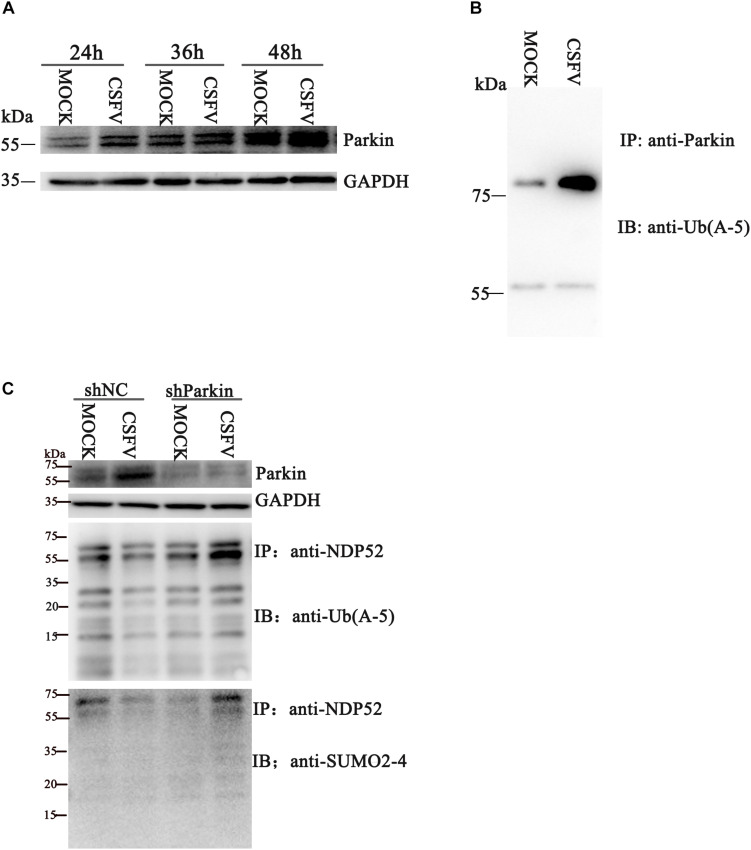
CSFV infection promotes ubiquitination of Parkin2 and mediates NDP52 modification via the PINK1-Parkin pathway. **(A)** PK-15 cells were treated as in Figure1 A. Western blotting was used to examine Parkin2, N^*pro*^ and GAPDH protein expression 24, 36, and 48 h after CSFV infection. **(B)** PK-15 cells were infected with CSFV (MOI = 0.1) for 24 h. Cell lysates were immunoprecipitated with an anti-Parkin antibody and assayed for Parkin2 with anti-Ub(A-5) antibody. **(C)** PK-15 cells were infected with CSFV (MOI = 0.1) for 24 h, followed by shParkin2 or shNC plasmid (MOI = 10) for 24 h. After immunoprecipitation with an anti-NDP52 antibody, NDP52 ubiquitination and SUMOylation were blotted with anti-Ub-5a and anti-SUMO2-4 antibodies.

Several reports suggest that NDP52 is closely related to the PINK1-Parkin pathway (Heo et al., 2015). To verify whether CSFV infection affects NDP52 modification via the PINK1-Parkin pathway, we first examined Parkin expression and ubiquitination at 24 h post-CSFV infection. We observed that CSFV infection promotes Parkin2 protein expression and ubiquitination (Figures 2A,B). The shParkin interference plasmid (Table 1) was applied to PK-15 cells for 24 h. Then, cells were infected with CSFV at an MOI of 0.1. Cell lysate was immunoprecipitated with anti-NDP52 antibody and immunoblotted with SUMO2-4 antibody or Ub(A-5) antibody. The results showed that ubiquitinated and SUMOylation NDP52 were significantly increased with shParkin, suggesting that Parkin plays a key role in NDP52 protein modification (Figure 2C). Thus, CSFV can activate the PINK1-Parkin pathway and promote the expression of Parkin. After inhibition of Parkin, the protein modification of NDP52 induced by CSFV is attenuated. These results show that CSFV infection promotes the ubiquitination of Parkin and mediates NDP52 modification via the PINK1-Parkin pathway.

### NDP52 Inhibition Decreases CSFV Replication

**FIGURE 3:**
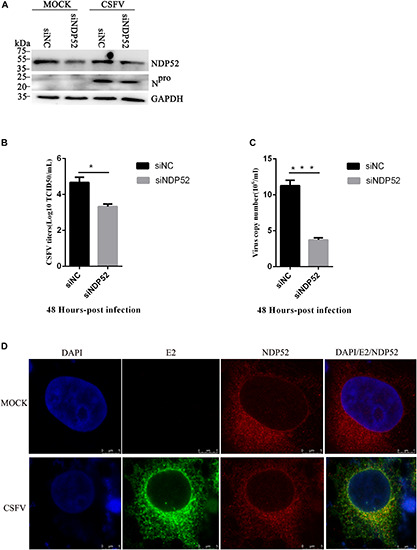
NDP52 inhibition reduces CSFV replication. **(A)** PK-15 cells were mock-infected or infected with CSFV (MOI = 0.1) after transfection with siNDP52 for 24 h. NDP52 expression was analyzed by Western blotting at 24 hpi. CSFV infection was verified by immunoblotting with anti-CSFV N^*pro*^ antibody. GAPDH was used as an internal loading control. **(B)** Statistical analysis of the effect of NDP52 siRNA transfection on the viral copy numbers in CSFV-infected cells. PK-15 cells were transfected with scrambled or NDP52 siRNA for 24 h, followed by mock infection and CSFV infection (MOI = 0.1). At 48 hpi, CSFV RNA levels were analyzed by real-time qRT-PCR (mean ± SD; *n* = 3; ^∗^*P* < 0.05; ^∗∗^*P* < 0.01; ^∗∗∗^*P* < 0.001; ^#^*P* > 0.05). *P*-values were calculated using an unpaired Student’s *t*-test. **(C)** Statistical analysis of the effect of NDP52 siRNA transfection on virus titers in CSFV-infected cells. PK-15 cells were transfected with scrambled or NDP52 siRNA for 24 h, followed by mock infection and CSFV infection (MOI = 0.1). At 48 hpi, CSFV titers were analyzed (mean ± SD; *n* = 3; ^∗^*P* < 0.05; ^∗∗^*P* < 0.01; ^∗∗∗^*P* < 0.001; ^#^*P* ¿ 0.05). *P*-values were calculated using an unpaired Student’s *t*-test. **(D)** PK-15 cells were mock-infected or infected with CSFV (MOI = 0.1), after 24 h infection, cells were immunostained with antibodies against CSFV E2 (green) and NDP52 (red). In the merged images, protein colocalization is displayed as yellow.

To assess the functional effects of NDP52 on CSFV infection, we performed RNA knockdown experiments to silence endogenous NDP52 expression in CSFV-infected PK-15 cells. As shown in Figure 3A, the silencing effect on NDP52 expression was verified by western blotting. Decreased expression of CSFV N^*pro*^ protein in NDP52-silenced cells suggested that CSFV replication is promoted by NDP52. To verify this finding, we analyzed CSFV replication after treatment with NDP52 siRNA, by measuring viral titers and RNA copy numbers. These results indicated that NDP52 silencing decreased viral replication in PK-15 cells (Figures 3B,C), demonstrating a positive role for NDP52 in CSFV replication. Moreover, we found that CSFV E2 protein and NDP52 colocalize to the cytoplasm by confocal microscopy (Figure 3D). The above results indicated that NDP52 promotes replication of CSFV and can colocalize with CSFV structural proteins.

### NDP52 Inhibition Reduces Autophagy Related Proteins Expression, but Decreases Ubiquitin and LC3 Binding

**FIGURE 4:**
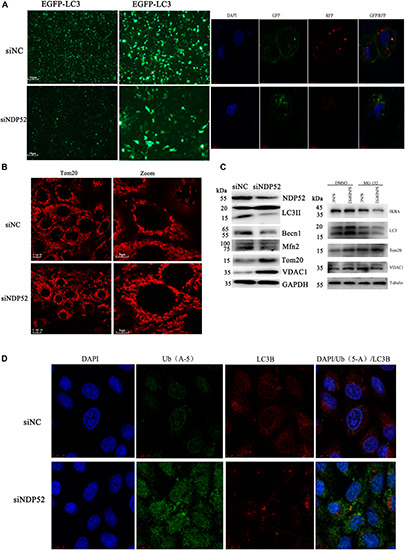
Inhibition of NDP52 reduces autophagy and mitophagy, and decreases ubiquitin and LC3 binding. **(A)** PK-15 cells were transfected with scrambled or NDP52 siRNA for 24 h. Then, cells were transfected with EGFP-LC3 or GFP-RFP-LC3 plasmid or immunostained with antibodies against LC3 (red). After NDP52 inhibition, eGFP levels were significantly reduced, the yellow dots were more than red dots and the LC3B expression in the cytoplasm. **(B)** PK-15 cells were treated as above, after 24 h transfection, cells were immunostained with antibodies against Tom20 (red). After NDP52 inhibition, Tom20 expression was enhanced. **(C)** The expression of the autophagy markers, LC3 and Beclin-I, and mitophagy markers, Mfn2, VDAC, and Tom20, in PK-15 cells were analyzed by Western blotting after transfection with scrambled or NDP52 siRNA for 24 h. GAPDH was used as the internal loading control. The expression of IκB-a, LC3, VDAC, and Tom20 in PK-15 cells were analyzed by Western blotting after co-transfection with scrambled or NDP52 siRNA with DMSO or MG-132 for 24 h. Tubulin was used as the internal loading control. **(D)** Ub(A-5) (green) and LC3 (red) expression in PK-15 cells. In the merged images, protein colocalization is displayed as yellow.

Several studies show that NDP52 is essential for autophagosome maturation (Tumbarello et al., 2012; Verlhac et al., 2015b). To investigate the role of NDP52 in CSFV-mediated autophagy, we examined the effect of NDP52 on autophagy and mitophagy. We assessed expression of the autophagy marker LC3 by transfecting the plasmid of GFP-LC3 and observed by fluorescence microscopy (Figure 4A). We also used the plasmid of GFP-RFP-LC3 to detect its distribution in cells. The results showed that LC3B in the cytoplasm was reduced and the yellow spots more than red spots in NDP52-inhibited cells. Thus, inhibition of NDP52 reduced LC3 expression and the normal fusion of autophagosomes with lysosomes. We also assessed the mitochondrial autophagy marker Tom20 using laser confocal microscopy and found that inhibition of NDP52 enhanced Tom20 expression (Figure 4B), suggesting positive regulation of autophagy by NDP52. At the same time, we detected expression of cellular and mitochondrial autophagy markers LC3B and Beclin-1 expression decreased, while VDAC1 and Tom20 expression increased (Figure 4C), indicating that inhibition of NDP52 has an inhibitory effect on the expression of autophagy proteins. To explore the role of ubiquitin in this process, we co-transfected NDP52 interfering RNA with MG-132 (5 μM), a ubiquitin-proteasome system inhibitor. We found that in the cells treated with MG-132, the effect of SiNDP52 on the autophagy-related protein disappeared, indicated that ubiquitin-proteasome system affects the effect of NDP52 on autophagy. In selective autophagy, the function of NDP52 is closely related to LC3 and ubiquitin, so we examined the expression and colocalization of LC3 and Ub(A-5) in cells. Indeed, NDP52 inhibition also decreased LC3 expression and subsequent binding to Ub(A-5) (Figure 4D).

### Inhibition of NDP52 Reduces Colocalization of Autophagy Vesicle CD63 With CSFV E2 Protein

**FIGURE 5:**
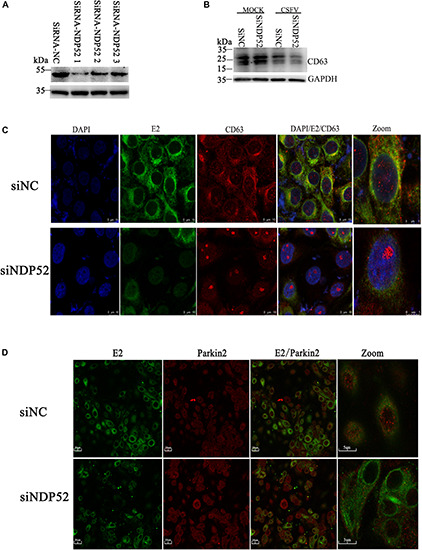
Inhibition of NDP52 reduces CD63 and Ub(A-5) binding to E2. **(A)** Three different NDP52 interference plasmids were verified by Western blot. GAPDH was used as the internal loading control. siRNA-NDP52-1 was used to inhibit NDP52 expression for subsequent experiments. **(B)** PK-15 cells were transfected with scrambled or NDP52 siRNA for 24 h, followed by mock or CSFV infection (MOI = 0.1). CD63 protein expression was detected by Western blot. GAPDH was used as the internal loading control. **(C)** Treated PK-15 cells were immunostained with antibodies against CSFV E2 (green) and CD63 (red). **(D)** PK-15 cells were immunostained with antibodies against CSFV E2 (green) and Ub(A-5) (red).

Our previous studies showed that CSFV induces autophagy, with autophagosomes as self-replication sites. However, the specific mechanism of CSFV-autophagosome entry is unclear (Pei et al., 2013). Because NDP52 regulates autophagy, we investigated the role of NDP52 in autophagic vesicles mediated by CSFV infection. We screened NDP52-interfering RNAs using western blotting and found that SiNDP52-1 had the best interference effect (Figure 5A). After NDP52 RNA interference for 24 h, PK-15 cells were infected with CSFV or mock-infected. Western blots showed that NDP52 inhibition decreased expression of CD63, a marker of autophagic vesicles (Figure 5B). Moreover, in the case of CSFV infection, this inhibition was more pronounced, indicating that NDP52 has a positive effect on the expression of CD63. To further verify the effect of NDP52 on CD63 and E2 colocalization, we used laser confocal microscopy to observe the localization of CD63. The results showed that CD63 binding to the CSFV E2 protein was decreased in the cytoplasm after NDP52 suppression (Figure 5C). We also found that Ub-5a binding to the CSFV E2 protein was reduced after NDP52 suppression (Figure 5D), suggesting that NDP52 plays an import role in CSFV entry into autophagosomes. These results indicate that NDP52 promotes colocalization of E2 with CD63 and Ub(A-5).

### Inhibition of NDP52 Activates the NF-κB Pathway to Release IFN/TNF Cytokines

**FIGURE 6:**
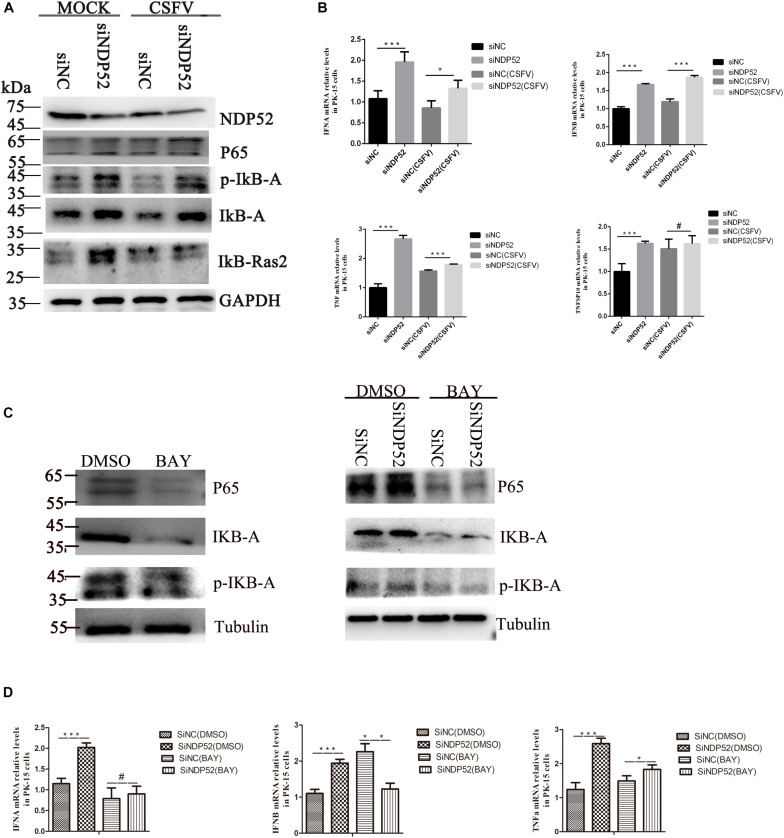
Inhibition of NDP52 promotes innate immunity through the NF-κB signaling pathway. **(A)** Inhibition of NDP52 has a positive effect on NF-κB signaling pathway. PK-15 cells were mock-infected or infected with CSFV (MOI = 0.1) after transfection with siNDP52 for 24 h. NF-κB signaling pathway proteins IκB-A, P65, IκB-Ras expression were analyzed by western blotting at 24 hpi. **(B)** PK-15 cells were treated as above. mRNA levels of the cytokines IFN-α, IFN-β, and TNF were detected by qRT-PCR. **(C)** PK-15 cells were co-transfection with scrambled or NDP52 siRNA with DMSO or BAY (5 μM) for 24 h. NF-κB signaling pathway proteins IκB-A, P65, p-IκB-A expression were analyzed by western blotting at 24 hpi. **(D)** PK-15 cells were treated as **(C)**, mRNA levels of the cytokines IFN-α, IFN-β, and TNF were detected by qRT-PCR. The data represent mean ± SD in three independent experiments. Data were tested by one-way ANOVA with *post hoc* LSD (L). ^∗^*P* < 0.05; ^∗∗^*P* < 0.01; ^∗∗∗^*P* < 0.001; #*P* > 0.05.

NF-κB activation during viral infection is part of the protective response to pathogens (Santoro et al., 2003). Interestingly, CSFV replication in cells suppresses type I IFN-inducible antiviral activity and apoptosis by interfering with IFN production, thereby resulting in the persistent survival of CSFV in host cells *in vitro* (Bensaude, 2004). To examine the effect of NDP52 on the innate antiviral immune pathway, we first examined NF-κB signaling activation following NDP52 inhibition. We observed increased expression of NF-κB signaling pathway proteins after NDP52 inhibition, indicating that inhibition of NDP52 promotes the activation of the NF-κB signaling pathway (Figure 6A). We also found that inhibition of NDP52 promotes release of cytokines, including IFN-α, IFN-β, and TNF (Figure 6B), indicating that NDP52 also plays an important role in the innate immune response. Therefore, NDP52 may inhibit CSFV infection via the NF-κB signaling pathway. In order to verify whether the release of SiNDP52 on the above cytokines is regulated by the NF-κB pathway, we treated cells with NF-κB inhibitor BAY 11-7082 (5 μM) and found that BAY 11-7082 had no effect on cytokine release, but BAY 11-7082 affects the effect of SiNDP52 on IFN-α release (Figures 6C,D).

### Cell Viability Was Not Affected by RNA Interference

**FIGURE 7:**
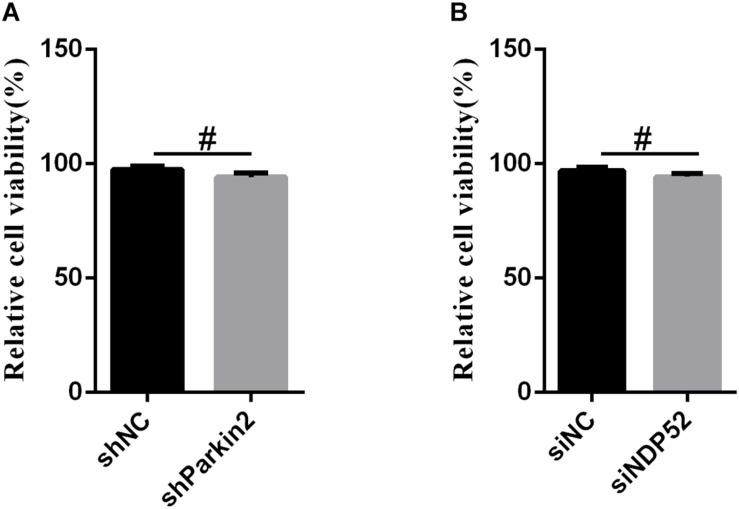
The effect of RNA interference on cell viability. The cell viability of PK-15 cells transfected with scrambled or Parkin shRNA **(A)** and siNC or NDP52 siRNA **(B)** was analyzed using the CCK8 assay as described in section “Materials and Methods” (mean ± SD; *n* = 3; ^∗^*P* < 0.05; ^∗∗^*P* < 0.01; ^∗∗∗^*P* < 0.001; ^#^*P* > 0.05).

To exclude the possibility that NDP52 siRNA inhibited CSFV replication or that Parkin shRNA inhibited NDP52 protein modification by downregulating cell viability, we assessed the effects of RNA interference on the viability of PK-15 cells. The results showed no significant changes in the viability of cells following silencing of NDP52 or Parkin (*P* > 0.05) (Figure 7).

## Discussion

The body has several cellular immune mechanisms to maintain the internal environment and prevent invasion by pathogenic microorganisms. However, many viruses have evolved replication strategies to cope with the adverse host environment. Indeed, virus-induced autophagy as a protective mechanism has been widely reported (Sugden et al., 2005). In our previous study, we found that CSFV infection induces complete and high-yield autophagic flux and that CSFV replicates in the autophagosome. Together, these processes provide a suitable environment for long-term viral infection in host cells. Further research found that the viral E2 protein colocalizes with the autophagosome marker CD63 (Metzelaars et al., 1991), but the function of this interaction was unclear. As an autophagy receptor, NDP52 plays an important role both in bacterial and viral infection. NDP52 mediates targeting of cytosolic substrates to autophagy machinery, and promotes the maturation of autophagic vesicles (Von muhlinen et al., 2012; Cemma et al., 2014; Leymarie et al., 2017). Therefore, we wanted to define the role of the autophagy receptor NDP52 in CSFV infection. The current study provides further insights into the role of NDP52 in CSFV-autophagic vesicle entry and the mechanism of induction of virus-mediated autophagy.

In the case of viral infection, Mohamud et al. (2018) reported that NDP52 binds to the coxsackievirus B3 viral protein VP1 and mediates VP1 ubiquitination via the K48 ubiquitin pathway. Other studies have shown that CALCOCO2 binds the non-structural protein 2 of the Chikungunya virus to promote viral replication in humans (Judith et al., 2013), and plays a pro-viral role in measles viral infection (Petkova et al., 2017). In this study, our results showed that CSFV infection inhibits NDP52 expression. Moreover, NDP52-interfering RNA inhibits CSFV replication, indicating that NDP52 plays an important role in the infection of swine fever virus.

During viral infection, autophagy receptors can induce viral phagocytosis in a ubiquitin-dependent manner and can bind directly to viral capsid proteins or indirectly to host ubiquitin factors via E3 ubiquitin ligase (Mohamud et al., 2018). In the present study, we found that CSFV infection can reduce NDP52 ubiquitination and SUMO2-4 protein modification. Since the PINK1-Parkin pathway and autophagy receptors are closely related to functional ubiquitin modification, we examined the CSFV-mediated regulation of NDP52 protein modification after Parkin inhibition. CSFV can mediate NDP52 ubiquitination by PINK1-Parkin. Interestingly, when we inhibited NDP52 expression by RNA interference, we found that Ub(A-5) expression increased while LC3 expression decreased. Ubiquitin acts as a signaling molecule, which causes polyubiquitination of autophagic substrates (Lu et al., 2017a, b). Autophagy receptor proteins recognize and bind autophagic substrates through a ubiquitin domain-dependent or independent manner (Walinda et al., 2014). The substrate is anchored to the autophagosome membrane by its LIR, followed by autophagosome-lysosome fusion and substrate degradation. We speculate polyubiquitination is required to recruit NDP52 or other autophagy receptors and reduce LC3 expression, and thus virus-induced ubiquitin molecules persist within the cell.

Because NDP52 mediates bacterial entry into autophagosomes (Sudhakar et al., 2019), we investigated whether NDP52 plays a role in CSFV entry into autophagosomes. We first assessed the effect of NDP52 on autophagy and observed that autophagy markers LC3 and Beclin-I decreased. Further analyses revealed that after NDP52 inhibition, CD63 protein expression was reduced, leading to decreased binding of CD63 to the viral E2 protein. These results suggest that NDP52 plays an important role in the mechanism of CSFV entry into autophagic vesicles. However, it is unclear whether NDP52 is modified by self-protein or by modifying CSFV protein to promote CSFV entry into autophagic vesicles.

NDP52 is involved in the negative regulation of the classical NF-κB pathway (Inomata et al., 2011). Our previous studies confirmed that CSFV can bind to MDA5 and RIG-1, inducing NF-κB nuclear translocation via RIG-1 signaling (Xiao-Ying et al., 2013). Moreover, NF-κB signaling also regulates CSFV replication (Ling et al., 2018). However, the effect of NDP52 on the NF-κB signaling pathway during CSFV infection was unclear. Therefore, we used RNA interference to inhibit NDP52 after CSFV infection. We found that NDP52 inhibition activates the NF-κB pathway, including P65, Iκb-Ras, IκBa, and p-IκBa. Furthermore, NDP52 inhibition promotes TNF and type I IFN release. CSFV infection attenuates this activation, suggesting that CSFV may have an inhibitory effect on the NF-κB signaling pathway.

In summary, our study provides the first evidence that CSFV infection regulates and modifies the autophagy receptor NDP52. Further, we uncovered the dual CD63 and NF-κB-induced mechanisms used by NDP52 to promote viral propagation.

## Data Availability Statement

All datasets generated for this study are included in the article/supplementary material.

## Author Contributions

SF, MiZ, KW, and JC conceived and designed the study. SF, KW, CL, MiZ, JC, EZ, and SM performed the experiments. SF, YC, MeZ, DS, and XL analyzed the data. SF, MiZ, KW, HD, JL, LY, and JC wrote the manuscript. All authors read and agreed upon the final manuscript.

## Conflict of Interest

The authors declare that the research was conducted in the absence of any commercial or financial relationships that could be construed as a potential conflict of interest.

## References

[B1] Becher P. Avalos Ramirez R. Orlich M. Cedillo Rosales S. König M. Schweizer M. (2003). Genetic and antigenic characterization of novel pestivirus genotypes: implications for classification. *Virology* 311 96–104. 10.1016/s0042-6822(03)00192-2 12832207

[B2] Bensaude E. (2004). Classical swine fever virus induces proinflammatory cytokines and tissue factor expression and inhibits apoptosis and interferon synthesis during the establishment of long-term infection of porcine vascular endothelial cells. *J. Gen. Virol.* 85 1029–1037. 10.1099/vir.0.19637-0 15039545

[B3] Bizargity P. Schröppel B. (2014). Autophagy: basic principles and relevance to transplant immunity. *Am. J. Transplant.* 14 1731–1739. 10.1111/ajt.12743 24934965

[B4] Cemma M. Kim P. K. Brumell J. H. (2014). The ubiquitin-binding adaptor proteins p62/SQSTM1 and NDP52 are recruited independently to bacteria-associated microdomains to target *Salmonella* to the autophagy pathway. *Autophagy* 7 341–345. 10.4161/auto.7.3.14046 21079414 PMC3060414

[B5] Ciechomska I. A. Gabrusiewicz K. Szczepankiewicz A. A. Kaminska B. (2013). Endoplasmic reticulum stress triggers autophagy. *Oncogene* 32 1518–1529. 10.1038/onc.2012.174 22580614

[B6] Deretic V. Saitoh T. Akira S. (2013). Autophagy in infection, inflammation and immunity. *Nat. Rev. Immunol.* 13 722–737. 10.1038/nri3532 24064518 PMC5340150

[B7] Fan S. Yuan J. Deng S. Chen Y. Xie B. Wu K. (2018). Activation of Interleukin-1β Release by the Classical Swine Fever Virus Is Dependent on the NLRP3 Inflammasome, Which Affects Virus Growth in Monocytes. *Front. Cell. Infect. Microbiol.* 8:225. 10.3389/fcimb.2018.00225 30013955 PMC6036178

[B8] Fu T. Liu J. Wang Y. Xie X. Hu S. Pan L. (2018). Mechanistic insights into the interactions of NAP1 with the SKICH domains of NDP52 and TAX1BP1. *Proc. Natl. Acad. Sci. U.S.A.* 115 E11651–E11660. 10.1073/pnas.1811421115 30459273 PMC6294882

[B9] Gou H. Zhao M. Fan S. Yuan J. Liao J. He W. (2017). Autophagy induces apoptosis and death of T lymphocytes in the spleen of pigs infected with CSFV. *Sci. Rep.* 7:13577. 10.1038/s41598-017-14082-9 29051589 PMC5648758

[B10] Heinz-Jurgen T. R. S. Emilie W. Tillmann R. Gregor M. (1991). Hog cholera virus_ molecular composition of virgins from a pestiferous. *J. Virol.* 65 4705–4712. 1870198 10.1128/jvi.65.9.4705-4712.1991 PMC248926

[B11] Heo J.-M. Ordureau A. Paulo J. A. Rinehart J. Harper J. W. (2015). The PINK11-PARKIN mitochondrial ubiquitylation pathway drives a program of OPTN/NDP52 recruitment and TBK1 activation to promote mitophagy. *Mol. Cell* 60 7–20. 10.1016/j.molcel.2015.08.016 26365381 PMC4592482

[B12] Hongchao G. Mingqiu Z. Hailuan X. Jin Y. Wencheng H. Mengjiao Z. (2017). CSFV induced mitochondrial fission and mitophagy to inhibit apoptosis. *Oncotarget* 24 39382–39400. 10.18632/oncotarget.17030 28455958 PMC5503620

[B13] Høyer-Hansen M. Jäättelä M. (2008). Autophagy An emerging target for cancer therapy. *Autophagy* 4 574–580. 10.4161/auto.5921 18362515

[B14] Inomata M. Niida S. Shibata K.-I. Into T. (2011). Regulation of Toll-like receptor signaling by NDP52-mediated selective autophagy is normally inactivated by A20. *Cell. Mol. Life Sci.* 69 963–979. 10.1007/s00018-011-0819-y 21964925 PMC3285758

[B15] Ivashkiv L. B. Donlin L. T. (2013). Regulation of type I interferon responses. *Nat. Rev. Immunol.* 14 36–49. 10.1038/nri3581 24362405 PMC4084561

[B16] Jin S. Tian S. Luo M. Xie W. Liu T. Duan T. (2017). Tetherin suppresses type I interferon signaling by targeting MAVS for NDP52-mediated selective autophagic degradation in human cells. *Mol. Cell* 68 308.e6–322.e6. 10.1016/j.molcel.2017.09.005 28965816

[B17] Johns H. L. Bensaude E. La Rocca S. A. Seago J. Charleston B. Steinbach F. (2009). Classical swine fever virus infection protects aortic endothelial cells from pIpC-mediated apoptosis. *J. Gen. Virol.* 91 1038–1046. 10.1099/vir.0.016576-0 20007358

[B18] Judith D. Mostowy S. Bourai M. Gangneux N. Lelek M. Lucas-Hourani M. (2013). Species-specific impact of the autophagy machinery on Chikungunya virus infection. *EMBO Rep.* 14 534–544. 10.1038/embor.2013.51 23619093 PMC3674439

[B19] Kocaturk N. M. Gozuacik D. (2018). Crosstalk between mammalian autophagy and the ubiquitin-proteasome system. *Front. Cell Dev. Biol.* 6:128. 10.3389/fcell.2018.00128 PMC6175981 30333975

[B20] Lazarou M. Sliter D. A. Kane L. A. Sarraf S. A. Wang C. Burman J. L. (2015). The ubiquitin kinase PINK11 recruits autophagy receptors to induce mitophagy. *Nature* 524 309–314. 10.1038/nature14893 26266977 PMC5018156

[B21] Leymarie O. Meyer L. Tafforeau L. Lotteau V. Costa B. D. Delmas B. (2017). Influenza virus protein PB1-F2 interacts with CALCOCO2 (NDP52) to modulate innate immune response. *J. Gen. Virol.* 98 1196–1208. 10.1099/jgv.0.000782 28613140

[B22] Ling S. Luo M. Jiang S. Liu J. Ding C. Zhang Q. (2018). Cellular Hsp27 interacts with classical swine fever virus NS5A protein and negatively regulates viral replication by the NF-κB signaling pathway. *Virology* 518 202–209. 10.1016/j.virol.2018.02.020 29525670

[B23] Liu T. Zhang L. Joo D. Sun S.-C. (2017). NF-κB signaling in inflammation. *Signal Transduct. Target. Ther.* 2:17023. 10.1038/sigtrans.2017.23 PMC5661633 29158945

[B24] Liu Z. Chen P. Gao H. Gu Y. Yang J. Peng H. (2014). Ubiquitylation of autophagy receptor optineurin by HACE1 activates selective autophagy for tumor suppression. *Cancer Cell* 26 106–120. 10.1016/j.ccr.2014.05.015 25026213 PMC4166492

[B25] Lu K. Den Brave F. Jentsch S. (2017a). Pathway choice between proteasomal and autophagic degradation. *Autophagy* 13 1799–1800. 10.1080/15548627.2017.1358851 28813181 PMC5965392

[B26] Lu K. Den Brave F. Jentsch S. (2017b). Receptor oligomerization guides pathway choice between proteasomal and autophagic degradation. *Nat. Cell Biol.* 19 732–739. 10.1038/ncb3531 28504708

[B27] Lum J. J. Bauer D. E. Kong M. Harris M. H. Li C. Lindsten T. (2005). Growth factor regulation of autophagy and cell survival in the absence of apoptosis. *Cell* 120 237–248. 10.1016/j.cell.2004.11.046 15680329

[B28] Majzoub K. Wrensch F. Baumert T. F. (2019). The innate antiviral response in animals: an evolutionary perspective from flagellates to humans. *Viruses* 11:758. 10.3390/v11080758 31426357 PMC6723221

[B29] Metzelaars M. J. Wijngaard P. L. Peter P. J. Sixma J. J. Nieuwenhuiss H. K. Cleversb H. C. (1991). CD63 antigen. A novel lysosomal membrane glycoprotein, cloned by a screening procedure for intracellular antigens in eukaryotic cells. *J. Biol. Chem.* 266 3239–3245. 1993697

[B30] Minowa-Nozawa A. Nozawa T. Okamoto-Furuta K. Kohda H. Nakagawa I. (2017). Rab35 GTPase recruits NDP52 to autophagy targets. *EMBO J.* 36 2790–2807. 10.15252/embj.201796463 28848034 PMC5599792

[B31] Mohamud Y. Luo H. (2018). The intertwined life cycles of enterovirus and autophagy. *Virulence* 10 470–480. 10.1080/21505594.2018.1551010 30475087 PMC6550542

[B32] Mohamud Y. Qu J. Xue Y. C. Liu H. Deng H. Luo H. (2018). CALCOCO2/NDP52 and SQSTM1/p62 differentially regulate coxsackievirus B3 propagation. *Cell Death Differ.* 26 1062–1076. 10.1038/s41418-018-0185-5 30154446 PMC6748094

[B33] Nakamura S. Yoshimori T. (2017). New insights into autophagosome–lysosome fusion. *J. Cell Sci.* 130 1209–1216. 10.1242/jcs.196352 28302910

[B34] Oeckinghaus A. Ghosh S. (2009). The NF- B family of transcription factors and its regulation. *Cold Spring Harb. Perspect. Biol.* 1:a000034. 10.1101/cshperspect.a000034 20066092 PMC2773619

[B35] Paton D. J. Greiser-Wilke I. (2003). Classical swine fever–an update. *Res. Vet. Sci.* 75 169–178. 10.1016/s0034-5288(03)00076-6 13129664

[B36] Pei J. Deng J. Ye Z. Wang J. Gou H. Liu W. (2016). Absence of autophagy promotes apoptosis by modulating the ROS-dependent RLR signaling pathway in classical swine fever virus-infected cells. *Autophagy* 12 1738–1758. 10.1080/15548627.2016.1196318 27463126 PMC5079672

[B37] Pei J. Zhao M. Ye Z. Gou H. Wang J. Yi L. (2013). Autophagy enhances the replication of classical swine fever virus in vitro. *Autophagy* 10 93–110. 10.4161/auto.26843 24262968 PMC4389882

[B38] Petkova D. Verlhac P. Rozières A. Baguet J. Claviere M. Kretz-Remy C. (2017). Distinct contributions of autophagy receptors in measles virus replication. *Viruses* 9:123. 10.3390/v9050123 28531150 PMC5454435

[B39] Ravenhill B. J. Boyle K. B. Von Muhlinen N. Ellison C. J. Masson G. R. Otten E. G. (2019). The cargo receptor NDP52 initiates selective autophagy by recruiting the ULK complex to cytosol-invading bacteria. *Mol. Cell* 74 320.e6–329.e6. 10.1016/j.molcel.2019.01.041 30853402 PMC6477152

[B40] Santoro M. G. Rossi A. Amici C. (2003). NF-κB and virus infection_ who controls whom. *EMBO J.* 22 2552–2560. 10.1093/emboj/cdg267 12773372 PMC156764

[B41] Scherz-Shouval R. Shvets E. Fass E. Shorer H. Gil L. Elazar Z. (2007). Reactive oxygen species are essential for autophagy and specifically regulate the activity of Atg. *EMBO J.* 26 1749–1760. 10.1038/sj.emboj.7601623 17347651 PMC1847657

[B42] Schmitz M. L. Kracht M. Saul V. V. (2014). The intricate interplay between RNA viruses and NF-κB. *Biochim. Biophys. Acta Mol. Cell Res.* 1843 2754–2764. 10.1016/j.bbamcr.2014.08.004 25116307 PMC7114235

[B43] Shaid S. Brandts C. H. Serve H. Dikic I. (2012). Ubiquitination and selective autophagy. *Cell Death Differ.* 20 21–30. 10.1038/cdd.2012.72 22722335 PMC3524631

[B44] Sharma V. Verma S. Seranova E. Sarkar S. Kumar D. (2018). Selective autophagy and xenophagy in infection and disease. *Front. Cell Dev. Biol.* 6:147. 10.3389/fcell.2018.00147 30483501 PMC6243101

[B45] Sudhakar P. Jacomin A.-C. Hautefort I. Samavedam S. Fatemian K. Ari E. (2019). Targeted interplay between bacterial pathogens and host autophagy. *Autophagy* 15 1620–1633. 10.1080/15548627.2019.1590519 30909843 PMC6693458

[B46] Sugden B. Jackson W. T. Giddings T. H. Taylor M. P. Mulinyawe S. Rabinovitch M. (2005). Subversion of cellular autophagosomal machinery by RNA viruses. *PLoS Biol.* 3:e156. 10.1371/journal.pbio.0030156 15884975 PMC1084330

[B47] Tattoli I. Sorbara M. T. Vuckovic D. Ling A. Soares F. Carneiro L. A. (2012). Amino acid starvation induced by invasive bacterial pathogens triggers an innate host defense program. *Cell Host Microbe* 11 563–575. 10.1016/j.chom.2012.04.012 22704617

[B48] Tumbarello D. A. Waxse B. J. Arden S. D. Bright N. A. Kendrick-Jones J. Buss F. (2012). Autophagy receptors link myosin VI to autophagosomes to mediate Tom1-dependent autophagosome maturation and fusion with the lysosome. *Nat. Cell Biol.* 14 1024–1035. 10.1038/ncb2589 23023224 PMC3472162

[B49] Verlhac P. Grégoire I. P. Azocar O. Petkova D. S. Baguet J. Viret C. (2015a). Autophagy receptor NDP52 regulates pathogen-containing autophagosome maturation. *Cell Host Microbe* 17 515–525. 10.1016/j.chom.2015.02.008 25771791

[B50] Verlhac P. Viret C. Faure M. (2015b). Dual function of CALCOCO2/NDP52 during xenophagy. *Autophagy* 11 965–966. 10.1080/15548627.2015.1046672 25998689 PMC4502821

[B51] Von muhlinen N. Akutsu M. Ravenhill B. J. Foeglein A. Bloor S. Rutherford T. J. (2012). LC3C, bound selectively by a noncanonical LIR motif in NDP52, is required for antibacterial autophagy. *Mol. Cell* 48 329–342. 10.1016/j.molcel.2012.08.024 23022382 PMC3510444

[B52] Walinda E. Morimoto D. Sugase K. Konuma T. Tochio H. Shirakawa M. (2014). Solution structure of the ubiquitin-associated (UBA) domain of human autophagy receptor NBR1 and its interaction with ubiquitin and polyubiquitin. *J. Biol. Chem.* 289 13890–13902. 10.1074/jbc.M114.555441 24692539 PMC4022861

[B53] Wang L. Ou J. J. (2018). Regulation of autophagy by Hepatitis C virus for its replication. *DNA Cell Biol.* 37 287–290. 10.1089/dna.2017.4115 29350547 PMC5963595

[B54] Xiao-Ying D. Wen-Jun L. Ming-Qiu Z. Jia-Ying W. Jing-Jing P. Yong-Wen L. (2013). Classical swine fever virus triggers RIG-I and MDA5-dependent signaling pathway to IRF-3 and NF-κB activation to promote secretion of interferon and inflammatory cytokines in porcine alveolar macrophages.*pdf*>. *Virol. J.* 10 1–11. 24034559 10.1186/1743-422X-10-286 PMC3849481

[B55] Zaffagnini G. Martens S. (2016). Mechanisms of selective autophagy. *J. Mol. Biol.* 428 1714–1724. 10.1016/j.jmb.2016.02.004 26876603 PMC4871809

