# Corrigendum: Dual NDP52 Function in Persistent CSFV Infection

**DOI:** 10.3389/fmicb.2021.673468

**Published:** 2021-04-12

**Authors:** Shuangqi Fan, Keke Wu, Chaowei Luo, Xin Li, Mengpo Zhao, Dan Song, Shengming Ma, Erpeng Zhu, Yuming Chen, Hongxing Ding, Lin Yi, Jun Li, Mingqiu Zhao, Jinding Chen

**Affiliations:** College of Veterinary Medicine, South China Agricultural University, Guangzhou, China

**Keywords:** classical swine fever virus, NDP52, ubiquitination, CD63, NF-κB

In the original article, there was an error. ^**^In paragraph 5 of the introduction, NDP52 is typed as NDP5: “In ***Salmonella typhimurium*** **infection, NDP5 promotes pathogen-containing autophagosome maturation and independently regulates targeting of bacteria to mature autophagosomes (Verlhac et al.**, **2015a**)”^**^.

A correction has been made to ^**^the introduction^**^, ^**^Paragraph 5^**^:

^**^In ***Salmonella typhimurium* infection, NDP52 promotes pathogen-containing autophagosome maturation and independently regulates targeting of bacteria to mature autophagosomes (Verlhac et al.**, **2015a**)^**^.

The authors apologize for this error and state that this does not change the scientific conclusions of the article in any way. The original article has been updated.
